# Peripartum cardiomyopathy and dilated cardiomyopathy: different at heart

**DOI:** 10.3389/fphys.2014.00531

**Published:** 2015-01-15

**Authors:** Ilse A. E. Bollen, Elza D. Van Deel, Diederik W. D. Kuster, Jolanda Van Der Velden

**Affiliations:** Department of Physiology, Institute for Cardiovascular Research (ICaR-VU), VU University Medical CenterAmsterdam, Netherlands

**Keywords:** dilated cardiomyopathy, peripartum cardiomyopathy, oxidative stress, prolactin, microvasculature, heart failure, titin

## Abstract

Peripartum cardiomyopathy (PPCM) is a severe cardiac disease occurring in the last month of pregnancy or in the first 5 months after delivery and shows many similar clinical characteristics as dilated cardiomyopathy (DCM) such as ventricle dilation and systolic dysfunction. While PPCM was believed to be DCM triggered by pregnancy, more and more studies show important differences between these diseases. While it is likely they share part of their pathogenesis such as increased oxidative stress and an impaired microvasculature, discrepancies seen in disease progression and outcome indicate there must be differences in pathogenesis as well. In this review, we compared studies in DCM and PPCM to search for overlapping and deviating disease etiology, pathogenesis and outcome in order to understand why these cardiomyopathies share similar clinical features but have different underlying pathologies.

## Introduction

Peripartum cardiomyopathy (PPCM) is a cardiac disease that can have many different clinical presentations but shares similar clinical symptoms with dilated cardiomyopathy (DCM) such as ventricle dilation, impaired systolic function and arrhythmias (Pearson et al., 2000; Elliott et al., 2008). Therefore, PPCM is often seen as DCM initiated during pregnancy. However, there are some important differences between the two cardiomyopathies which argue for separation of the two disease states. This review aims to compare studies performed in PPCM and DCM patients and animal models in order to better understand why these cardiomyopathies share similar clinical features but have different underlying pathomechanisms.

## Clinical presentations

Dilated cardiomyopathy has a variety of causes such as: genetic factors, viral infections, alcohol abuse and myocardial infarction. Current classification of DCM excludes cases in which coronary artery disease, loading conditions, hypertension, valvular diseases and congenital heart diseases could have caused the observed phenotype (Elliott et al., 2008). The exact prevalence of idiopathic DCM varies between studies, from 1:2700 in the original epidemiological study (Codd et al., 1989) to 1:250 in a recent study (Hershberger et al., 2013). PPCM, as the name implies, develops during the last month of pregnancy, during delivery or within the first 5 months after delivery (Pearson et al., 2000). The prevalence of PPCM has been established to be 1:4000 to 1:1000 live births in the Western world (Elkayam, 2011; Kolte et al., 2014), although there is regional variation, e.g. the prevalence of PPCM in Haiti is 1:300 live births (Fett et al., 2005). In contrast to DCM, which typically presents later in life (Hershberger et al., 2013), PPCM often develops in young, previously healthy women and is a disease which progresses quickly to cardiac dysfunction and failure as is evident in the high mortality and transplantation rate (Van Hoeven et al., 1993; Hilfiker-Kleiner et al., 2007; Sliwa et al., 2010; Elkayam, 2011; Fett, 2014). As symptoms of PPCM such as dyspnea, fatigue and exercise incapacity are similar to normal pregnancy symptoms, the disease is often diagnosed late. If diagnosed and treated early, women suffering from PPCM may be stabilized upon treatment with β-blockers, ACE inhibitors and bromocriptine (BR) and reversal of the phenotype resulting in favorable outcome and recovery is not uncommon (Van Hoeven et al., 1993; Felker et al., 2000; Hilfiker-Kleiner et al., 2007; Sliwa et al., 2010; Elkayam, 2011; Ballo et al., 2012; Haghikia et al., 2013; Fett, 2014). This is contrary to what is often observed in DCM which is characterized by a late onset and slow disease progression that might be stabilized but unlikely to be reversed upon treatment (Van Hoeven et al., 1993; Fish, 2012; Hershberger et al., 2013).

## Physiological adaptations during pregnancy

The pathogenesis of PPCM is tightly bound to the cardiac changes that accompany normal pregnancies. It is therefore important to understand the physiological changes that occur during pregnancy. The increased hemodynamic load caused by the increased blood volume results in physiological hypertrophy, in which chamber dimensions increase, concomitant with a proportional increase in wall thickness to cope with the increased hemodynamic load and to facilitate increased cardiac output (Clapp and Capeless, 1997; Umar et al., 2012). This physiological hypertrophy is different from the pathological (eccentric) hypertrophy seen in both PPCM and DCM in which chamber dimensions increase while wall thickness does not increase proportionally in response to increased hemodynamic load (Gaasch and Zile, 2011). According to the law of Laplace this will lead to increased wall stress. Physiological pregnancy-related hypertrophy is reversible post-partum, usually within 1 year after delivery (Clapp and Capeless, 1997). Other differences between pathological and physiological hypertrophy are the absence of fibrosis and the absence of induction of the fetal gene program in physiological hypertrophy (McMullen and Jennings, 2007; Umar et al., 2012). In addition, angiogenic factors such as vascular endothelial growth factor A (VEGF A) are secreted during pregnancy which facilitates myocardial angiogenesis in order to increase capillary density proportionally to cardiomyocyte size. Both capillary density as well as VEGF levels return to pre-pregnancy levels post-partum (Umar et al., 2012; Chung and Leinwand, 2014). Pregnancy is often seen as a cardiac stress model upon which underlying cardiac dysfunction may reveal itself due to the increased hemodynamic load on the heart and hormonal changes during pregnancy (Chung and Leinwand, 2014). However, the increase in hemodynamic load starts early in pregnancy (Clapp and Capeless, 1997) while PPCM develops in the last month of pregnancy or even post-partum. It is therefore unlikely that the increased hemodynamic load is the sole cause of PPCM.

## Genetic predisposition and titin isoform switching

As stated before, DCM can have a genetic cause, leading to a familial form of DCM. Many mutations have been linked to DCM. However, many of these genes are also implicated in hypertrophic and restrictive cardiomyopathies (Hershberger et al., 2013). A common gene which is mutated in 6% of DCM patients is the gene encoding for the protein lamin-A/C (*LMNA*) (Parks et al., 2008). *LMNA* mutations show a relatively high penetrance compared with mutations in other genes and patients carrying *LMNA* mutations often have conduction abnormalities (Parks et al., 2008; Hershberger et al., 2013). In addition, Herman et al. showed a high incidence of truncated variants in the gene encoding for the protein titin (*TTN*) in DCM patients (Herman et al., 2012). In PPCM, little is known about causative pathogenic mutations. However, the fact that multiple PPCM cases within one family have been reported (Van Spaendonck-Zwarts et al., 2014) suggests that gene mutations could play an important role. Furthermore, reports about DCM and PPCM cases within families and the identification of mutations (Morales et al., 2010; Van Spaendonck-Zwarts et al., 2010, 2014) strengthen the suggestion of a genetic cause and overlap in etiology of PPCM and DCM. A recent study showed a high incidence of *TTN* variants in PPCM patients, and this cohort was marked by slow recovery (Van Spaendonck-Zwarts et al., 2014). However, it has been proposed that *TTN* mutations are not always disease causing, but might act as disease modifiers as truncated *TTN* variants are present in 3% of the general population (Herman et al., 2012). Knowledge about pathogenic effects of gene mutations would enable the identification of persons at risk for the development of DCM and PPCM and thereby facilitate early diagnosis and treatment.

The protein titin acts as a multifunctional spring that can exist as two distinct isoforms in the adult human heart; a compliant N2BA isoform and a stiff N2B isoform. A shift to more N2BA titin isoform and subsequent reduced passive stiffness was shown in DCM patients previously (Makarenko et al., 2004; Nagueh et al., 2004). Apart from isoform shift, alterations in titin post-translational modifications such as phosphorylation are able to alter passive force development (Granzier and Labeit, 2002). Titin isoform has also been suggested to play a role in the ability of the heart to adapt contractility in response to stretch, known as the Frank-Starling mechanism (Fukuda et al., 2003). Unfortunately, limited data is available about the role of titin in PPCM, although increased compliant titin isoform and lowered passive tension has been reported in one PPCM patient with a *TTN* mutation (Van Spaendonck-Zwarts et al., 2014). Titin can also be modified under oxidizing conditions in which disulfide bridges can be formed in titin's N2B unique sequence possibly resulting in increased passive stiffness (Grützner et al., 2009). In addition, S-glutathionylation of cysteine residues in the Ig regions of titin under the influence of redox signaling has been suggested to lower passive stiffness (Alegre-Cebollada et al., 2014). As oxidative stress is present in both PPCM and DCM, as described later in this review, it is possible that this will also affect titin function although this has not been established *in vivo* yet.

## Oxidative stress and prolactin: a deadly combination

In both DCM and PPCM, oxidative stress is a key player in disease pathogenesis. However the exact consequences of reactive oxygen species (ROS) production differ notably between the two disease states as will be discussed below. In normal pregnancy, ROS production increases during the course of pregnancy and decreases post-partum to normal levels (Toescu et al., 2002). In an attempt to counterbalance the detrimental ROS production, total anti-oxidant capacity also increases during pregnancy and remains elevated post-partum (Toescu et al., 2002). In both PPCM animal models and human PPCM patients, oxidative stress levels are increased compared to healthy controls (Hilfiker-Kleiner et al., 2007). An explanation for the increased oxidative stress in PPCM can be found in the PPCM mouse model with cardiomyocyte restricted deletion of Signal transducer and activator of transcription 3 (STAT3) (Hilfiker-Kleiner et al., 2007). This transcription factor regulates the expression of the superoxide scavenger Manganese superoxide dismutase (MnSOD) (Negoro et al., 2001). Accordingly, in the cardiac STAT3 KO mice PPCM is accompanied by a reduced expression of MnSOD and concomitant oxidative stress (Hilfiker-Kleiner et al., 2007). A crucial pathway in PPCM that is instigated by elevated oxidative stress is the cleavage of the hormone prolactin (PRL) by ROS-activated Cathepsin D (CD) (Hilfiker-Kleiner et al., 2007). Upon ROS activation CD cleaves full-length prolactin (PRL) of 23 kDa into a smaller 16 kDa form which has detrimental effects on cardiomyocyte metabolism and the microvasculature (Hilfiker-Kleiner et al., 2007, 2012; Hilfiker-Kleiner and Sliwa, 2014). The idea that PRL plays a crucial role in PPCM is further strengthened by the fact that PRL levels rise at the end of pregnancy and remain high post-partum during breast feeding which coincides with the onset of PPCM (Grattan et al., 2008). Accordingly, injection of adenoviral vectors expressing 16 kDa PRL in non-pregnant mice led to the development of cardiac dysfunction, dilation of the left ventricle (LV) and decreased myocardial capillary density (Hilfiker-Kleiner et al., 2007). As decreased levels of STAT3, high levels of oxidative stress, high CD activity and 16 kDa PRL have also been observed in human PPCM patients (Hilfiker-Kleiner et al., 2007; Haghikia et al., 2013), it strengthens the suggestion that insufficient defense against oxidative stress and subsequent formation of 16 kDa PRL plays an important role in PPCM pathogenesis. The compound BR is able to inhibit PRL release from the pituitary gland. Blockade of PRL by BR treatment in PPCM patients has been shown to improve cardiac function and increase survival, although only a limited number of studies containing small patient cohorts have been performed so far (Hilfiker-Kleiner et al., 2007; Sliwa et al., 2010; Ballo et al., 2012; Haghikia et al., 2013). Together these results indicate that cleavage of PRL under oxidative stress is key in the disease development in both human PPCM as well as the cardiac STAT-3 KO PPCM mouse-model (Hilfiker-Kleiner et al., 2007). However, not all patients treated with BR recover (Sliwa et al., 2010; Haghikia et al., 2013). Therefore, even though the PRL-mediated pathway is probably a major determinant in this disease, other pathways are likely to play a role. Since STAT3 signaling has many different functions, it is unlikely that the repressed MnSOD expression alone explains all observations in the STAT3 KO mouse model. Additionally, in heterozygous MnSOD^+/−^ mice a reduction of MnSOD levels of 50% causes cardiac alterations under basal conditions (Remmen et al., 2001) and induces cardiac remodeling after pregnancy (Hilfiker-Kleiner et al., 2007), but it does not lead to the dilated PPCM phenotype often seen in PPCM patients and the cardiac STAT3 KO mouse model. This implies that besides MnSOD additional factors are essential for the development of PPCM.

STATs are activated upon phosphorylation by Janus kinases (JAK) and are multifunctional transcription factors. In cardiomyocytes STAT3 is involved in survival, sarcomere integrity, cell growth and ROS production (Haghikia et al., 2014). In the vasculature STAT3 promotes vascularization through stimulation of VEGF signaling (Osugi et al., 2002). Clearly STAT3 affects multiple cardiac processes besides generation of the 16 kDa PRL (Haghikia et al., 2014). In the cardiac STAT3 KO model, low STAT3 levels have been associated with the up-regulation of miR-199 (Haghikia et al., 2011). MiR-199 down-regulates specific ubiquitin conjugating enzymes thereby affecting the ubiquitin proteasome system (UPS). In an *in vitro* model, this disturbance of the UPS resulted in decreased protein levels of myosin heavy chain and troponin T, thereby causing disruption of sarcomere structure (Haghikia et al., 2011). Also in DCM patients, low STAT3 levels, increased miR-199 levels and decreased levels of ubiquitin conjugating enzymes have been observed (Haghikia et al., 2011). Low STAT3 levels have been observed in both PPCM (Hilfiker-Kleiner et al., 2007) and DCM patients (Podewski, 2003), indicating STAT3 may be part of the common pathway of both diseases. However, it should be noted that MnSOD protein levels and activity have been reported to be unchanged in DCM patients compared to non-failing controls in the myocardium (Dieterich et al., 2000) and a higher MnSOD activity was observed in serum (Wojciechowska et al., 2014). This indicates decreased STAT3 levels might have different downstream effects in DCM and PPCM and loss of MnSOD might form an etiology-specific (additional) source of oxidative stress in PPCM.

**Figure 1:**
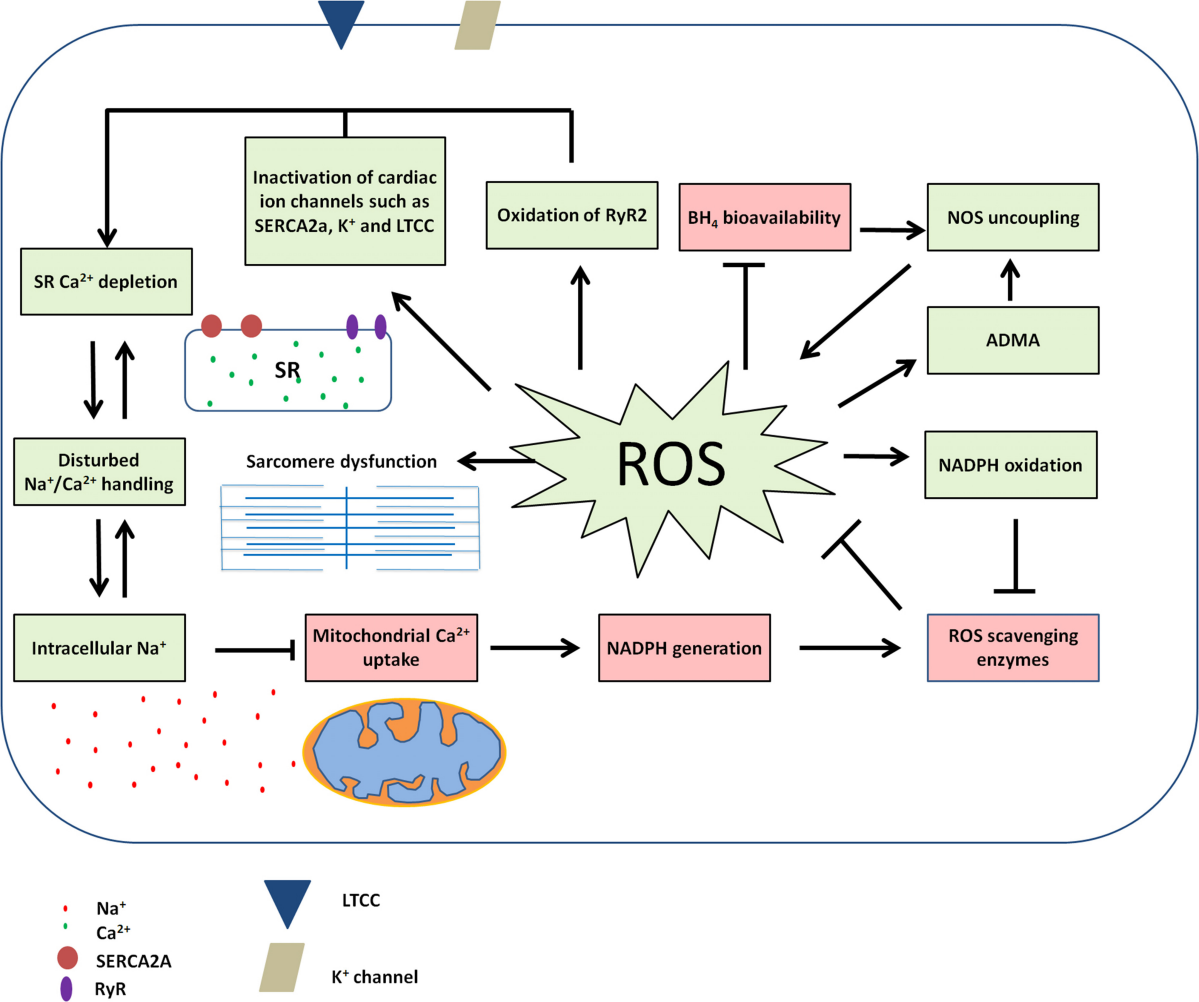
**Disturbed Na^+^/Ca^2+^ signaling and ROS production can be locked in a vicious circle in heart failure**. Red boxes indicate down-regulated or disturbed factors; green boxes indicate up-regulated factors in disease.

Oxidative stress is observed in many forms of heart failure (HF), including PPCM and DCM, and disturbs various pathways. However, the high levels of PRL during the final stages of pregnancy combined with high oxidative stress, likely make PPCM develop at such a high pace. As PRL levels are many fold lower in non-pregnant and non-nursing individuals (Grattan et al., 2008), it is unlikely that PRL also plays a role in DCM patients. Although oxidative stress has many detrimental effects in the failing heart it should be noted that ROS also fulfill a beneficial role in physiological circumstances. For example, NADPH oxidase (NOXs) produce ROS in a tightly regulated manner and play an important role in stretch-induced contraction by sensitization of the ryanodine receptor (RyR2) through oxidation (Prosser et al., 2011). However, in DCM NOX activity is up-regulated leading to increased ROS production (Maack et al., 2003). Amongst others oxidative stress inactivates important ion channels such as the L-type-calcium channel (LTCC) and the sarcoendoplasmic reticulum calcium transport ATPase (SERCA2a) and, in combination with disturbed RyR2 sensitization, may lead to Ca^2+^ depletion from the sarcoplasmic reticulum (Sag et al., 2013). Another general feature of HF is elevated cytosolic Na^+^ levels (Despa, 2002) that result in decreased Ca^2+^ uptake and consequently reduced NADPH generation in mitochondria [28]. This disturbed balance of ions within cardiomyocytes causes cardiac dysfunction due to disturbed excitation-contraction coupling (Sag et al., 2013). In turn reduced mitochondrial NADPH availability leads to reduced generation of mitochondrial antioxidants and increased levels of ROS (Kohlhaas et al., 2010). In addition, ROS causes NADPH oxidation, thereby further reducing NADPH bioavailability for the production of ROS scavenger enzymes. In addition, ROS can reduce the bioavailability of tetrahydrobiopterin (BH_4_) and thereby induce the uncoupling of nitric oxide synthase (NOS). As a result NOS no longer produces beneficial nitric oxide (NO) molecules, but itself becomes a producer of superoxide (Verhaar, 2004). In this way ROS production can be self-sustaining and cause additional ROS production (Seddon et al., 2007) thereby locking the heart in a vicious circle of oxidative stress (Kohlhaas et al., 2010). Similarly the NOS inhibitor asymmetric dimethylarginine (ADMA), a factor shown to be up-regulated in both DCM and PPCM and described later in this review, has been suggested to stimulate NOS-uncoupling mediated ROS production (Wilcox, 2012). However, it should be noted that ADMA levels at expected pathological concentrations only had modest effect on ROS production in this study (Druhan et al., 2008). The vicious circle of ROS production amplification and Na^+^/Ca^2+^ imbalance can be visualized in Figure 1. Apart from affecting cardiomyocyte ion balance, ROS has many other detrimental effects on cardiomyocytes such as nitration of creatine kinase, SERCA2a, voltage gated K^+^ channels, desmin, myosin heavy chain and α-actinin, induction of DNA damage, cell death and fibrosis (Pacher et al., 2007; Seddon et al., 2007). As fibrosis can increase myocardial stiffness and does not contribute to active force development, increased fibrosis can influence both systolic and diastolic function. Increased fibrosis is common in DCM patients (Assomull et al., 2006; Herpel et al., 2006) and the cardiac STAT3 KO mouse model of PPCM (Ricke-Hoch et al., 2014), however, there are limited and contradictory reports about fibrosis in PPCM patients (Kawano et al., 2008; Mouquet et al., 2008; Leurent et al., 2009; Ntusi and Chin, 2009).

Together, alterations induced by ROS can lead to cardiomyocyte dysfunction through impaired Na^+^ and Ca^2+^ handling, impaired sarcomere integrity, cardiomyocyte death and fibrosis formation. Although 16 kDa PRL is an important contributor to the pathophysiology of PPCM, it is likely that oxidative stress-induced cardiac pathology in PPCM also involve other ROS induced pathways.

## Disturbed sarcomere integrity and angiogenic imbalance

The previously described disturbance in the ubiquitin proteasome system (UPS) system also leads to increased levels of ADMA (Haghikia et al., 2011), a factor involved in reducing NO availability by competing with the NOS substrate L-arginine, and increasing ROS production by endothelial cells thereby contributing to the amplification of ROS production and cardiac dysfunction shown in Figure 1 (Druhan et al., 2008). Also in human PPCM (Haghikia et al., 2013) and DCM patients increased levels of ADMA have been found and in DCM decreased L-arginine/ADMA ratio was shown to be a predictor of mortality (Anderssohn et al., 2012). The male mice of the cardiac STAT3 KO model showed age-related loss of capillary density and a DCM-like phenotype upon aging including fibrosis and impaired sarcomere organization (Hilfiker-Kleiner et al., 2004). Several studies indicate that damaged microvasculature or disturbance in pro- and anti-angiogenic factors play a significant role in PPCM pathogenesis. A study by Patten et al. showed a high prevalence (33%) of (pre)eclampsia in their PPCM cohort (Patten et al., 2012), which is believed to be a risk factor for PPCM. It has been shown that PPCM leads to capillary dissociation and endothelial cell apoptosis (Hilfiker-Kleiner et al., 2007). Patten et al. suggested that the anti-angiogenic state might worsen the severity of the disease (Patten et al., 2012). In late pregnancy, anti-angiogenic factors, such as soluble, fms-like tyrosine kinase 1 (sFLT1), are secreted and thereby inhibit the increased VEGF signaling during pregnancy (Chung and Leinwand, 2014). The increase in anti-angiogenic factors is significantly higher in patients with (pre)eclampsia which results in decreased VEGF levels (Levine et al., 2004) and also unusually high levels of sFLT1 have been observed in PPCM patients (Patten et al., 2012). This indicates the anti-angiogenic state is important in both (pre)eclampsia and PPCM pathogenesis and might explain the high prevalence of (pre)eclampsia among PPCM patients. A common finding in PPCM studies is a reduced capillary density in the post-partum phase (Hilfiker-Kleiner et al., 2007; Patten et al., 2012). Disturbed VEGF signaling and decreased capillary density have also been observed in DCM (Abraham et al., 2000; Karch et al., 2005; Lionetti et al., 2014). Another KO mouse model which lacks the cardiac PPARγ coactivator-1α (PGC-1α), also results in the development of PPCM (Patten et al., 2012). PGC-1α promotes angiogenesis by stimulating VEGF signaling (Arany et al., 2008; Patten et al., 2012), (cardiac) mitochondrial biogenesis and metabolism in a tissue specific manner (Lehman et al., 2000; Arany et al., 2005). However, also in this model PPCM development occurs through the MnSOD, ROS and 16 kDa PRL pathway as the phenotype could only be partly rescued by pro-angiogenic therapy in the form of VEGF A and treatment with both VEGF A and BR was needed to obtain full recovery of LV function. Indeed, in PGC-1α KO mice MnSOD was reduced and ROS increased, which is not surprising as PGC-1α is an inducer of MnSOD (Patten et al., 2012). Repressed levels of PGC-1α have also been observed in PPCM patients (Patten et al., 2012). PGC-1α could also be implied in DCM as PGC-1α KO mice showed inability to increase heart rate in response to dobutamine, had markedly depressed levels of ATP in cardiac muscle and showed ventricle dilation and reduced fractional shortening upon aging (7–8 months), indicating PGC-1α deficiency can induce a DCM-like phenotype (Arany et al., 2005). However, depressed levels of PGC-1α-regulated genes, but not expression levels of PGC-1α itself, have been reported in DCM (Sihag et al., 2009). The extend of depression of PGC-1α regulated genes has been shown to be correlated to LV systolic function, which indicate mitochondrial function as regulated by PGC-1α is impaired in HF (Sihag et al., 2009). The 16 kDa PRL increases NFκB activity induced by miR-146 and thereby causes endothelial cell apoptosis (Tabruyn et al., 2003; Halkein et al., 2013). In addition, 16 kDa PRL up-regulates exosome loading with miR-146 of endothelial cells. Uptake of these exosomes by cardiomyocytes causes miR-146 induced impaired metabolic activity (Halkein et al., 2013). An interesting notion is that miR-146 has been shown to be up-regulated in PPCM but not in DCM patients (Haghikia et al., 2013; Halkein et al., 2013). This indicates that although both patient groups suffer from dysfunctional microvasculature, the exact underlying pathogenesis might not be the same. Impaired microvasculature may result in insufficient oxygen and nutrient delivery to cardiomyocytes resulting in increased susceptibility to apoptosis and impaired cardiac function. It is clear that PPCM and DCM can result in similar clinical presentation even without the presence of PRL. The cleaved form of PRL is likely to cause an additional hit in PPCM quickly worsening disease progression and outcome. An overview of the changes in PPCM and DCM compared to healthy controls, grouped by downstream effect, can be seen in Table 1.

**Table 1:** **Altered parameters in PPCM and DCM compared to healthy controls**.

	**Parameter**	**PPCM**	**DCM**
Structure	Chamber dilation	↑	↑
	Sarcomere mutations	Possible	Possible
	Sarcomere integrity	↓	↓
	Fibrosis	↑ in animals, unknown in human	↑
	Fetal gene program	Unknown	↑
	Titin N2BA/N2B ratio	Unknown	↑
Oxidative stress	Oxidative stress	↑	↑
	MnSOD	↓	=
	16 kDa PRL	↑	Unlikely
	PGC-1α	↓	↓ target gene expression
	STAT3	↓	↓
Vasculature	Capillary density	↓	↓
	VEGF	↓	↓
	miR-146	↑	=
	miR-199	↑	↑
	ADMA	↑	↑

*References for the shown parameters are indicated in the text*.

## Pregnancy in DCM patients and subsequent pregnancies in PPCM patients

Given the similarities between PPCM and DCM, DCM patients presenting with the wish to conceive are a complicated patient group to counsel. Only a few studies have explored the disease alterations in DCM patients who become pregnant. The effects of pregnancy on cardiac function in DCM patients are contradictory as limited adverse events and recovery toward pre-pregnancy cardiac function (Bernstein and Magriples, 2001; Blatt et al., 2010), but also significant decreased cardiac event-free survival compared to non-pregnant DCM patients has been reported (Grewal et al., 2009). Adverse outcome in pregnant DCM patients was related to high NTproBNP levels, a known indicator for cardiac dysfunction, Blatt et al. (2010) and New York Heart Association functional classification class III or IV (Grewal et al., 2009). Most cardiac adverse events occurred at the beginning of the third trimester, which coincided with an increase in hemodynamic load on the heart (Grewal et al., 2009) while PRL levels are still relatively low. Another case report that showed adverse events during pregnancy in a DCM patient also showed adverse events earlier in pregnancy than is observed in PPCM (Gevaert et al., 2014). Therefore, most adverse events seen in pregnant DCM patients are most likely due to the cardiac stress during pregnancy that is being placed on an already troubled heart. However, most adverse events were successfully treated and recovery to pre-pregnancy cardiac function was often established. A retrospective study by Bernstein et al. showed pregnancy is well-tolerated in stable DCM patients, while maternal outcome was significantly worse in PPCM patients (Bernstein and Magriples, 2001). Therefore, Bernstein et al. suggest the prognosis of PPCM patients should not be used when a stable DCM patient presents herself with the wish to conceive (Bernstein and Magriples, 2001). This strengthens the suggestion that although PPCM and DCM may share similar genetic predispositions and some common disease pathways, PPCM should not be considered as DCM occurring during or soon after pregnancy. However, it is also possible that pregnant DCM patients are protected from the fast deterioration of cardiac function seen in PPCM due to the fact that DCM patients are already treated for heart failure prior to and during pregnancy and are closely monitored. A small study by Hilfiker-Kleiner et al. showed BR treatment of PPCM patients in their subsequent pregnancy was able to improve survival and prevent relapse compared to PPCM patients without BR treatment (Hilfiker-Kleiner et al., 2007). Studies exploring subsequent pregnancies of PPCM patients revealed a HF relapse in 20–30% and the ability to recover was related to cardiac function after PPCM index pregnancy and prior to the subsequent pregnancy (Elkayam et al., 2005; Fett et al., 2010). From these studies it can be concluded that stable DCM patients are unlikely to develop the fast PPCM disease progression and that the outcome of the DCM patient group is often more favorable than that of PPCM patients. The potential uneventful previous and subsequent pregnancies in PPCM patients indicate we do not know all triggers of PPCM since any predisposing mutation and PRL would also have been present in previous and subsequent event-free pregnancies. As PRL is present in both pregnant DCM patients and PPCM patients, we cannot simply state that PPCM is triggered by the presence of PRL in combination with a weakened heart.

## Future perspectives

### Large cohort studies PPCM

At the moment there is still a lack of prospective studies exploring PPCM in larger cohorts. Most studies to date are case studies or small (often retrospective) cohort studies. A large, multicenter, randomized controlled clinical trial to evaluate BR treatment in 60 PPCM patients is ongoing, and the results should shed light on the effectiveness of BR treatment and outcome (US National Library of Medicine, 2012). In addition, the EURObservational Registry on PPCM has started to enroll patients that will be followed in a 1-year prospective study which will help to evaluate PPCM clinical presentations, risk factors, management and the effects on offspring worldwide (Sliwa et al., 2014).

### Genetic predispositions

Information about disease initiation and predispositions such as pathogenic mutations is still limited in PPCM and DCM. Therefore, more knowledge is needed on which mutations predispose people to DCM and PPCM and how these mutations exert their pathogenic effect in disease initiation and progression. Identification of these mutations would greatly enhance the ability to identify persons at risk to develop PPCM and DCM and by monitoring these patients early diagnosis and treatment could be facilitated thereby enhancing survival.

### Cardiac remodeling

As cardiac deterioration, heart transplantations and death often occur within a few months after diagnosis in PPCM, it is possible that the cardiac remodeling to cope with the new cardiac state and demands in PPCM is different than in a more slow progressive disease as DCM. For example, in heart failure it has been shown that the fetal gene program is initiated resulting in cardiac remodeling in order to compensate for decreased cardiac function, although it contributes to cardiac dysfunction at a later stage (Thum et al., 2007). The limited time for adaptation of the heart might be detrimental in PPCM patients, as the lack of time to mount a compensatory response causes the need for cardiac transplantation, or lead to death soon after diagnosis. On the other hand, full recovery of PPCM patients may be explained by lack of permanent cardiac remodeling. However, not enough is known about remodeling of the PPCM heart and the recommendation to not use gadolinium in pregnant women has limited the use of magnetic resonance imaging (MRI) to assess fibrosis in PPCM patients. Therefore, other techniques to assess cardiac remodeling such as immunohistochemical staining of biopsies or explanted heart tissue should be explored in order to shed light on cardiac remodeling in PPCM.

### Oxidative stress

Based on observations in the STAT3 mouse model, mitigation of the intracellular defense mechanism against oxidative stress in human PPCM so far mainly focused on the reduction of MnSOD levels. Indeed reduction of MnSOD levels is an important contributor to elevated ROS levels in PPCM patients. However, to further unravel the patho-mechanism of oxidative stress in PPCM, future studies should involve the potential contribution of other enzymatic and non-enzymatic ROS scavengers as well as that of potential sources of ROS. Reduced MnSOD expression and PRL cleavage as a result of concomitant oxidative stress have been identified as important regulators of PPCM. Still the complete etiology of PPCM is far from completely understood.

## Summary and conclusion

PPCM and DCM likely share part of their pathogenesis such as predisposing mutations, increased oxidative stress, an impaired microvasculature and damaged sarcomere integrity. However, the exact underlying pathways might be differently altered in PPCM and DCM. While 16 kDa PRL is likely to be a key player in PPCM it is unlikely to play a significant role in DCM. It is this 16 kDa PRL that might explain the faster deterioration of cardiac function in PPCM by inducing an additional cascade of cardiovascular impairment. However, given the observation that pregnant DCM patients do better than PPCM patients, this difference in PRL levels between PPCM patients and non-pregnant DCM patients cannot explain all differences observed. The reports about uneventful subsequent pregnancies in PPCM patients indicate additional causative factors, such as insufficient defense mechanisms against oxidative stress or a vulnerable microvasculature. Therefore, despite their overlap in disease etiology and clinical presentation, differences in underlying pathways, disease progression and outcome argue for separation of the two disease states.

### Conflict of interest statement

The authors declare that the research was conducted in the absence of any commercial or financial relationships that could be construed as a potential conflict of interest.

## Abbreviations

ADMA: Asymmetric dimethylarginine
BR: Bromocriptine
CD: Cathepsin D
DCM: Dilated cardiomyopathy
EF: Ejection Fraction
HF: Heart Failure
KO: Knock out
*LMNA*: Gene encoding the protein lamin-A/C
LV: Left ventricle
MnSOD: Manganese superoxide dismutase
NOS: Nitric oxide synthase
NOX: NADPH oxidase
PGC-1α: PPARγ coactivator-1α
PPCM: Peripartum cardiomyopathy
PRL: Prolactin
ROS: Reactive oxygen species
sFLT1: fms-like tyrosine kinase 1
STAT3: Signal transducer and activator of transcription 3
*TTN*: Gene encoding the protein titin
UPS: Ubiquitin proteasome system
VEGF: Vascular endothelial growth factor.
